# Adiposity markers and lung function in smokers: a cross-sectional study in a Mediterranean population

**DOI:** 10.1186/s12890-016-0341-y

**Published:** 2016-12-09

**Authors:** Mar Sorlí-Aguilar, Francisco Martín-Luján, Gemma Flores-Mateo, Cristina Jardí-Piñana, Estefania Aparicio-Llopis, Josep Basora-Gallisà, Rosa Solà-Alberich, for the ESPITAP Study Group investigators, G. Aguirre-Alava, M. Altamiras-Badia, E. Alvarez-Soler, C. Anguera-Perpiña, V. Arnau-Adan, M. Baiges-Folch, J. Basora-Gallisa, P. Berenguer-Atrio, A. Bibiloni-Sole, J. Blade-Creixenti, J. Blanch-Aubia, A. Boada-Tous, A. Borras-Gavalda, D. Borras-Vicente, J. J. Cabre-Vila, P. Camos-Guijosa, J. J. Canalejo-Escudero, G. Cando-Guasch, M. J. Castellar-Salinas, R. Castro-Pamies, L. Comino-Sillero, S. Dalmau-Vidal, M. J. DeAndres-DePablo, J. DelPozo-Nubio, A. Diego-Ferrer, J. M. Duran-Visiedo, T. Elviro-Bodoy, J. Ferrater-Cubells, J. Ferre-Gras, I Fustero-Fustero, R. Garcia-Aguila, C. Garcia-Gonzalo, A. Garcia-Masso, M. Gens-Barbera, S. Gil-Mancha, M. D Gil-Sanchez, C. Giner-Aguilo, J. M. Giro-Guasch, R. Girona-Real, F. Gomez-Santidrian, C. Grau-Perez, M. Grive-Isern, N. Guinjoan-Aymemi, J. M. Hernandez-Anguera, E. Hernandez-Lazaro, N. Hernandez-Vidal, A. Isach-Subirana, M. D. Jovani-Puig, M. Juncosa-Cabre, A. Lara-Pedrosa, M. T. Lara-Pedrosa, J. Ledo-Garcia, M. Lluis-Burgeño, A. Lorente-Zozaya, M. Mangrane-Ferrando, C. Mangrane-Guillen, J. Marimon-Barba, E. Marti-Suau, A. Martín-Lorente, N. Martin-Vergara, M. T. Martinez-Blesa, T. Martinez-Perez, R. Mas-Escoda, M. Medina-Clemente, M. Mengual-Miralles, N. Mora-Guilabert, A. Moreno-Lagunas, Y. Ortega-Vila, E. Oya-Girona, L. Palacios-Llamazares, M. I. Palma-Jimenez, J. Pardo-Andujar, I. Pascual-Palacios, M. L. Pelleja-Pellicer, M. Perez-Bauer, E. Perez-Galvez, T. Pineda-Rigau, J. L. Piñol-Moreso, A. Poca-Pastor, A. Prats-Caellas, R. Profitos-Amiell, A. Reche-Martinez, V. Revuelta-Garrido, C. Rey-Reñones, M. Ribes-Arganuy, A. Riera-Sole, B. Rius-Fernandez, C. Rubio-Gascon, J. Sabate-Mestre, R. Sagarra-Alamo, I. Sanchez-Oro, E. Sardaña-Alvarez, N. Sarra-Manetas, Y. Sarre-Torra, A. R. Silva-Orjuela, P. Soler-Barreras, R. Solis-Narvaez, E. Subirats-Sanz, R. Subirats-Segarra, M. Tersa-Alcobe, M. Timon-Torres, A. Urbaneja-Diez, O. Vazquez-Martinez, O. Vers-Lopez, M. Vila-Molet, R. V. Vila-Rodrigo, J. Vizcaino-Marin

**Affiliations:** 1 Study Group on Respiratory Tract Diseases (GEPAR), Institut Universitari d’Investigació en Atenció Primària Jordi Gol (IDIAP Jordi Gol), Barcelona, Spain; 2 Primary Healthcare Research Support Unit Tarragona-Reus, Institut Universitari d’Investigació en Atenció Primària Jordi Gol (IDIAP Jordi Gol), Reus, Spain; 3 School of Medicine and Health Sciences, Universitat Rovira i Virgili, Tarragona, Spain; 4 CAP Sant Pere - Institut Català de la Salut, C/Camí de Riudoms, 53-55, Reus, 43203 Tarragona Spain; 5 CIBERobn Physiopathology of Obesity and Nutrition, Institute of Health Carlos III (ISCIII), Madrid, Spain; 6 NFOC group School of Medicine and Health Sciences, Universitat Rovira i Virgili, Tarragona, Spain

**Keywords:** Lung function test, Overweight, Obesity, Waist circumference, Tobacco smoke

## Abstract

**Background:**

The aim of this study was to assess the association of key adiposity markers with lung function in smokers without respiratory disease in a Mediterranean population.

**Methods:**

We performed a cross-sectional study with baseline data from a representative sample of the ESPITAP study in Spain. Participants were 738 smokers (52.3% men) without respiratory disease, aged 35 to 70, selected from 12 primary health care centres. We assessed weight, height, body mass index (BMI), waist circumference (WC) and waist-to-height ratio (WHtR). The pulmonary functional parameters were forced vital capacity (FVC), forced expiratory volume in 1 s (FEV1) and FEV1/FVC ratio.

**Results:**

In this cohort of smokers, 22.2% of individuals had central obesity. FVC% was inversely associated with all anthropometric measures (BMI, WC and WHtR) in the overall population and in men; in women, only BMI was associated with FVC%. FEV1% was inversely associated to BMI and WC in the overall population, and to all anthropometric measures in men. Furthermore, both BMI and obesity were positively associated with FEV1/FVC ratio overall and when stratified by sex; this suggests a restrictive pattern explained by the altered ventilator mechanics experienced by people with obesity.

**Conclusion:**

In a Mediterranean population of smokers without respiratory symptoms, abdominal obesity, evaluated not only by BMI and WC but also WHtR, is inversely associated with lung function. Fat distribution appears more strongly related to pulmonary function parameters in men than in women. In smokers with high values for WC, WHtR and BMI, assessment of lung function is recommended.

**Trial registration:**

Current Controlled Trials NCT01194596. Registered 2 September 2010.

## Background

Smoking is an established cause of diseases and is responsible for most of the avoidable deaths in smokers due to cardiovascular diseases, respiratory diseases and cancer [1]. Tobacco smokers have reduced lung function, characterized by decreased forced expiratory volume after one second (FEV1) and forced vital capacity (FVC) in diagnostic tests, and smoking has been associated with environmental risks, genetic disorders, respiratory infections, poor dietary habits and obesity [2]. However, other factors such as body weight can exert an influence on lung function [3]. Specifically, excess weight has a negative impact on the respiratory system due to its effect on gas exchange, respiratory mechanics, muscular endurance and breath control [4, 5]. No consensus exists about the physiopathological mechanisms by which excess weight leads to respiratory complications, although it seems that these include mechanical impact on the diaphragm (impeding descent into the abdominal cavity) or on the chest wall (changes in compliance, the work of breathing and elastic recoil) [6].

Respiratory complications have been consistently reported in patients with obesity, a chronic disease characterized by the excessive accumulation of body fat and associated with a reduction in lung volume. Body fat can be measured using the body mass index (BMI) and classified into categories according to World Health Organization criteria [7]. The role of BMI relative to the risk of impaired lung function has been well studied. The most consistent effect is an exponential decrease in FEV1% and in functional residual capacity with increasing BMI [8–11]. On the other hand, a low BMI is associated with increased risk of mortality and is considered a negative prognostic factor for survival based on the degree of lung dysfunction [6, 12].

Nevertheless, studies using weight and BMI as the single relevant measurements of adiposity while ignoring other aspects of body composition, such as visceral fat or fat distribution, may miss the true dose–response curve between the distribution of adiposity and increased risk of disease or all-cause mortality. A recent systematic review and meta-analysis of the impact of adiposity distribution has clearly shown a significant inverse relationship between waist circumference (WC) and pulmonary function, with a greater effect size in men [13]. Furthermore, waist-to-height ratio (WHtR) and other indexes of fat distribution have been suggested to better identify high-risk subjects of different pathologies [14, 15]. WHtR has the benefit of adjusting WC according to height, a measurement that remains quite unchanged in adults; this reinforces the importance of changes in WC measurement. These newer indexes have even replaced BMI in several definitions for clinical diagnosis of metabolic syndrome that consider fat distribution a more accurate predictor of diabetes of cardiovascular disease [16]; however, they are not widely used in studies of respiratory function or diseases.

The aim of the present study was to assess the association between body weight, new indexes of fat distribution and lung function in a Mediterranean population of smokers with no diagnosis of respiratory disease.

## Methods

### Study design

A cross-sectional study was performed with baseline data from a representative sample of the ESPITAP study (Spanish acronym for “Effectiveness of Smoking Cessation Advice Combined with Spirometric Results in Adult Smokers”). This multicentre, randomized, clinical trial aimed to evaluate the effectiveness in the primary care setting of a structured motivational intervention and feedback on spirometry data to achieve smoking cessation, compared with usual clinical practice and assessed with respect to quit rates at 12 months after the intervention. The detailed protocol of the ESPITAP study has been previously published [17].

### Study subjects

Of the 195,343 patients aged 18 years and older from 12 primary care practices in the province of Tarragona (Spain) managed by the Catalan Health Institute who were randomized to the ESPITAP study groups, 738 were selected. Candidates for participation were smokers who visited a centre for any reason during regular office hours, met all inclusion criteria and none of the exclusion criteria (detailed below), and signed informed consent.

#### Inclusion criteria

Aged 35 to 70 years, current smoker (defined as having smoked daily during the past month, regardless of the quantity), cumulative consumption of more than 10 pack-years (defined as the daily average of cigarettes smoked, multiplied by the number of years of smoking, divided by 20 cigarettes in a pack). *Exclusion criteria*: any evidence of previous diagnosis of a respiratory disease, functional pulmonary testing conducted within the previous 12 months, presence of any chronic or terminal condition that would affect the baseline parameters or complicate the testing and analysis to be conducted during the study period, impossibility of completing follow-up for any reason, or patient refusal to participate in the study.

### Measurements

The baseline examination included a structured questionnaire designed to collect the necessary data: sociodemographic, history of diseases, medications and symptoms, alcohol consumption (standard drink unit/week), physical activity (hours/week), current daily smoking habit (cigarettes per day) and accumulated consumption (lifetime tobacco exposure in pack-years).

#### Lung function

Spirometry and bronchodilator tests were administered using a Datospir-600 ultrasound pneumotachograph (SIBELMED, S.A.), following a standardized procedure according to current recommendations [18]. The following criteria were used to determine normal pneumotachography values: FEV1 ≥ 80% of the predicted value, FVC ≥ 80% of the predicted value, and FEV1/FVC ≥ 0.7 [19].

#### Anthropometry and body composition

During a physical examination, height (m) and weight (kg) were measured with the participant in light clothing and no shoes, using calibrated scales and a wall-mounted stadiometer, respectively; BMI was calculated as the weight (kilograms) divided by the square of the height (meters). World Health Organization criteria were used to classify the population according to weight status (normal weight, BMI <25.0 kg/m^2^; overweight, BMI 25.0–29.9 kg/m^2^ and obesity, BMI ≥30.0 kg/m^2^) [7]; WC was measured midway between the lowest rib and the iliac crest using an anthropometric tape; the WHtR was calculated as WC divided by height, both in centimetres.

### Sample size

Accepting an alpha risk of 0.05 in a two-sided test with 243 subjects in the first group (normal weight) and 175 in the second group (obesity), the statistical power was greater than 99% to recognize as statistically significant a difference of means (97.9 of FVC% in normal-weight group and 87.8 of FVC% in obesity group). Moreover, accepting an alpha risk of 0.05 in a two-sided test with 243 subjects in the first group (normal weight) and 303 in the second group (overweight), the statistical power was 98% to recognize as statistically significant a difference of means (97.9 of FVC% in normal weight group and 92.8 of FVC% in overweight group).

### Statistical analysis

Quantitative variables were expressed as mean and standard deviation (SD) or median and range depending on the normal distribution of variable. We evaluated the association of categories of body weight and fat distribution measures with lung function according to the spirometry results. First, a Pearson correlation was performed to investigate association between weight, height, BMI, WC and WHtR and FVC%, FEV1% and FEV1/FVC ratio. Later, a multivariate linear regression analysis was performed for all participants and for men and women separately, applying the Full Maximum likelihood method of estimation. BMI <25 kg/m^2^ was used as reference value.

We applied Holm-Bonferroni corrections for multiple testing to *p*-values resulting from the Pearson correlation [20].

All statistical tests were 2-sided at the 5% significance level. Analyses were carried out using the Stata/MP 14.1 version (Stata Corp).

## Results

A total of 738 participants (52.3% men) were included. The baseline characteristics of the study participants are shown in Table 1. There were no significant differences between men and women in age, sociodemographic data, medical history or BMI, though men tended to be taller and heavier than women. Approximately 41% of the study population was overweight and 24% was obese, with higher values in men. The mean (SD) age of smoking onset was lower in men (17.10 ± 4.73 years vs. 18.71 ± 6.44 for women) and women smoked less (17.63 ± 9.30 cigarettes a day vs. 20.72 ± 11.94 for men). FVC%, FEV1 % and FEV1/FVC ratio were lower in men.

**Table 1:** Characteristics and lung function measures of the study sample, overall and separately for men and women

Variables	Men (*n* = 386)	Women (*n* = 352)	All (*n* = 738)
Sociodemographic			
Age, years	52.87 ± 8.23	48.90 ± 7.39	50.98 ± 8.08
Marital status, n (%)			
Married	299 (77.5)	226 (64.2)	525 (71.1)
Widower	3.0 (0.8)	19 (5.4)	22 (3.0)
Single	46 (12.0)	39 (11.1)	85 (11.6)
Separated/Divorced	35 (9.1)	66 (18.9)	101 (13.8)
Social class^a^, n (%)			
High	62 (16.1)	51 (14.5)	113 (15.4)
Medium	176 (46.0)	148 (42.0)	324 (43.9)
Low	145 (37.6)	151 (42.9)	296 (40.1)
Medical history, n (%)			
Diabetes mellitus	49 (12.7)	18 (5.1)	67 (9.1)
Dyslipidaemia	115 (29.8)	64 (18.2)	179 (24.3)
Hypertension	112 (29.0)	68 (19.3)	180 (24.4)
Cardiovascular disease	18 (4.7)	4 (1.1)	22 (3.0)
Central obesity	72 (18.6)	92 (26.1)	164 (22.2)
Health habits			
Alcohol consumption, standard drink/week	7 (0–9)	0 (0–2)	1 (0–9)
Physical activity, hours/week	2.42 ± 0.26	2.20 ± 0.19	2.31 ± 0.16
Smoking			
Start smoking age, years	17.10 ± 4.73	18.71 ± 6.44	17.87 ± 5.65
Current consumption, cigarettes/day	20.72 ± 11.94	17.63 ± 9.30	19.25 ± 10.87
Cumulative consumption, pack-years	36.77 ± 23.55	26.96 ± 16.62	32.09 ± 21.10
Anthropometric and body composition			
Weight, kg	80.38 ± 13.46	66.82 ± 14.15	73.92 ± 15.36
Height, cm	170.30 ± 6.93	158.35 ± 6.79	164.61 ± 9.09
BMI, kg/m^2^	27.63 ± 0.22	26.65 ± 0.28	27.16 ± 0.17
BMI categorization^b^, n (%)			
< 25.0 kg/m^2^	90 (23.3)	153 (44.5)	243 (32.9)
25.0–29.9 kg/m^2^	184 (47.7)	119 (33.8)	303 (41.1)
≥ 30.0 kg/m^2^	102 (26.4)	73 (21.2)	175 (23.7)
Waist circumference, cm	98.75 ± 10.44	92.27 ± 16.55	96.0 ± 13.73
Waist-to-height ratio	0.58 ± 0.06	0.59 ± 0.10	0.58 ± 0.82
Lung function parameters			
FVC, % of predicted	89.14 ± 0.84	98.10 ± 0.77	93.49 ± 0.59
FEV1, % of predicted	90.50 ± 0.96	99.73 ± 0.82	94.93 ± 0.66
FEV1/FVC ratio (%)	76.13 ± 0.40	78.59 ± 0.35	77.32 ± 0.27

Data are presented as number of patients (%) or mean ± standard deviation or median ± range depending on the type of variable

*BMI* Body mass index, *SBP* Systolic blood pressure, *DBP* Diastolic blood pressure, *ppm* Parts per million, *FVC* Forced vital capacity, *FEV1* Maximum expiratory volume in the first second of a forced exhalation

^a^According to the National Occupational Classification proposed by the Spanish Society of Epidemiology and the Spanish Society of Family and Community Medicine [41]

^b^According to the WHO classifications of body weight [7]

The correlation between the anthropometric measures and lung function are shown in Table 2. FVC % was inversely correlated with body weight (*r* = −0.203), BMI (*r* = −0.236), WC (*r* = −0.267) and WHtR (*r* = −0.261), but only in men. Furthermore, FEV1% was associated with WC (*r* = −0.226) and WHtR (*r* = −0.218) only in men. No association was found between FEV1/FVC ratio and the adiposity measures (BMI, WC and WHtR).

**Table 2:** Correlation between lung function and anthropometric parameters, overall and separately for men and women

Variables	Weight	Height	BMI	Waist circumference	Waist-to-height ratio
FVC % Predicted					
All	−0.315**	−0.203**	−0.243**	−0.261**	−0.196**
Women	−0.241**	−0.079	−0.215**	−0.159	−0.184
Men	−0.203**	0.024	−0.236**	−0.267**	−0.261**
FEV1 % Predicted					
All	−0.238**	−0.201**	−0.153**	−0.209**	−0.132
Women	−0.151*	−0.089	−0.115	−0.087	−0.087
Men	−0.134	−0.001	−0.148	−0.226**	−0.218**
FEV1/FVC ratio					
All	0.030	−0.137**	0.122	0.011	0.074
Women	0.121	−0.073	0.163	0.127	0.173
Men	0.120	−0.001	0.131	0.001	−0.018

Values represent the correlation coefficients. *p < 0.05, **p < 0.001

*BMI*, body mass index, *FVC* forced vital capacity, *FEV1* maximum expiratory volume in the first second of a forced exhalation

A multivariate linear regression analysis of lung function parameters and anthropometric measures, overall and separately for men and women, are shown in Table 3. FVC% was inversely and significantly associated with all anthropometric measures in the overall population (WHtR, *p =* 0.002; WC, *p <*0.001; continuous BMI, *p <*0.001; BMI ≥30, *p <*0.001) and men (WHtR, *p =* 0.001; WC, *p <*0.001; continuous BMI, *p <*0.001; BMI ≥30, *p <*0.001). By contrast, only continuous BMI was inversely associated with FVC% in women (*p =* 0.016). Likewise, FEV1% was inversely associated only with WC and continuous BMI in the overall population (*p =* 0.005 and *p =* 0.024, respectively), but with all anthropometric measures in men (WHtR, *p =* 0.007; WC, *p =* 0.002; continuous BMI, *p =* 0.029; BMI ≥30, *p =* 0.054). In women, none of the anthropometric indices was significantly associated with FEV1%. Finally, FEV1/FVC ratio was positively associated with BMI categorization and continuous BMI in the overall population and in men and women when analysed separately.

**Table 3:** Multivariate linear regression analysis of lung function and anthropometric parameters, overall and separately for men and women

Variables	Male			Female			All		
	β	95% CI	*p* values	β	95% CI	*p* values	β	95% CI	*p* values
FVC % Predicted									
Waist-to-height	−61.48	−98.00, −24,96	0.001	−14.76	−36.94, 7.41	0.190	−26.8	−46.61, −6.93	0.002
Waist circumference	−0.39	−0.60, −0.18	<0.001	−0.07	−0.21 0.07	0.328	−0.22	−0.34, −0.10	<0.001
BMI, continuous	−0.79	−1.19, −0.39	<0.001	−0.37	−0.67, −0.07	0.016	−0.59	−0.83, −0.34	<0.001
BMI categorization^a^									
< 25.0 kg/m^2^	Ref.	Ref.	Ref.	Ref.	Ref.	Ref.	Ref.	Ref.	Ref.
25.0–29.9 kg/m^2^	−4.67	−8.67, −0.66	0.023	−1.51	−4.94, 1.92	0.388	−4.21	−6.81, −1.60	0.002
≥ 30.0 kg/m^2^	−9.19	−13.82, −4.54	<0.001	−3.21	−7.45, 1.04	0.138	−67.25	−10.38, −4.13	<0.001
FEV1 % Predicted									
Waist-to-height	−59.91	−102.94, −16.88	0.007	−2.99	−27.32, 21.34	0.808	−16.94	−39.77, 5.88	0.145
Waist circumference	−0.40	−0.65, −0.15	0.002	−0.02	−0.17, 0.13	0.787	−0.20	−0.33, −0.06	0.005
BMI, continuous	−0.52	−0.99, −0.05	0.029	−0.10	−0.41, 0.22	0.547	−0.32	−0.59, −0.04	0.024
BMI categorization^a^									
< 25.0 kg/m^2^	Ref.	Ref.	Ref.	Ref.	Ref.	Ref.	Ref.	Ref.	Ref.
25.0–29.9 kg/m^2^	−0.78	−5.52, 3.96	0.609	1.43	−2.18, 5.04	0.436	−1.01	−3.95, 1.93	0.502
≥ 30.0 kg/m^2^	−5.39	−10.87, 0.99	0.054	0.08	−4.38, 4.54	0.971	−2.77	−6.25, 0.70	0.118
FEV1/FVC ratio									
Waist-to-height	1.13	−17.93, 20.20	0.907	11.58	1.46, 21.70	0.025	9.58	−0.16, 19.32	0.054
Waist circumference	0.01	−0.10, 0.12	0.917	0.05	−0.01, 0.12	0.113	0.02	−0.04, 0.08	0.512
BMI, continuous	0.24	0.11, 0.38	<0.001	0.31	0.11, 0.50	0.002	0.26	0.14, 0.37	<0.001
BMI categorization^a^									
< 25.0 kg/m^2^	Ref.	Ref.	Ref.	Ref.	Ref.	Ref.	Ref.	Ref.	Ref.
25.0–29.9 kg/m^2^	3.76	1.79, 5.73	<0.001	2.23	0.71, 3.75	0.004	2.43	1.20, 3.65	<0.001
≥ 30.0 kg/m^2^	3.81	1.53, 6.09	0.001	3.10	1.17, 4.93	0.002	2.89	1.42, 4.36	<0.001

Adjusted by social class, smoking cumulative consumption, physical activity, alcohol consumption, medical history hypertension, diabetes mellitus and dyslipidemia

*FVC* Forced vital capacity, *FEV1* Maximum expiratory volume in the first second of a forced exhalation; β: regression coefficient for each exposure variable, *CI* Confidence interval, *BMI* Body mass index

^a^According to the WHO classifications of body weight: normal weight (<25.0 kg/m^2^), overweight (25.0–29.9 kg/m^2^) and obese (≥30.0 kg/m^2^) [7]

## Discussion

This study, conducted in a Mediterranean population of smokers without pulmonary disease, showed that overweight, obesity and pattern of body fat distribution are inversely related to lung function. A positive association was found between FEV1/FVC ratio and BMI, overweight and obesity categories in both sexes. Moreover, a negative correlation was found between BMI, WC and WHtR and both FVC% and FEV1% in all smokers, but especially in men. These new adiposity markers provide evidence from a Mediterranean population of smokers and complement the findings of previous cross-sectional and prospective studies in other populations showing that an excess of adipose tissue and its distribution pattern are negatively related to pulmonary function, a basic indicator of respiratory health [8, 9, 21, 22].

The present study has both limitations and strengths to consider. Cross-sectional analysis was used to assess the ability of adiposity marker measures to predict a pulmonary function disorder, making inference of causality difficult. Further longitudinal analysis will provide stronger evidence of these associations. Smoking status, which has a detrimental effect on the lungs, is a potential confounding factor in the relationship between BMI, WC, WHtR and pulmonary function. Furthermore, our results pertain to a specific cohort of adult smokers (aged 35–70 years), a population with a high risk of lung disease [2]. The relationship between smoking and worse lung function is no longer subject to debate, given the available epidemiological, morphological and genetic evidence. However, more recent studies are demonstrating the importance of additional factors such as abdominal adiposity markers [6]. It is possible that impaired lung function parameters were better associated with WC or WHtR than with BMI because smokers tend to have a lower BMI [23]. On the other hand, BMI is the only measure of obesity reported in several other population-based studies [8, 24]. Therefore, a strength of our work is that few studies have evaluated the association between WHtR and lung function.

Our study showed that highly specific markers of increased abdominal adiposity such as WC and/or WHtR, already proposed as better adiposity indicators than BMI [13, 25], were associated with lower FVC% and FEV1% values. Furthermore, a recent meta-analysis supports the use of WC as a pulmonary risk indicator because high WC values are associated with pulmonary dysfunction [13]. Results of the present study also support the use of WHtR as a new adiposity distribution marker involved in pulmonary function, amplifying the hypothesis previously tested for cardiovascular diseases [26]. Those authors recommend using WHtR, the correction of WC according to the height of the individual, because this measurement remains quite unchanged in adults, which reinforces the importance of changes in WC measurement. WHtR has been inversely associated with cardiovascular risk [25], and now also with lung function in the present study.

In our analysis stratified by sex, the inverse association of WC and WHtR with impaired lung function (FEV1% and FVC %) was apparent in men but not in women. This finding is consistent with results from several other studies [8, 11, 27, 28], and supports the hypothesis that a sex-related difference in the pattern of fat distribution is one of the explanations for the sex difference in lung function impairment. Nonetheless, other studies have shown the opposite results. For example, in a cohort of patients with metabolic syndrome, pulmonary function was significantly lower in women than in men [29]. Although it was unclear why sex would be associated with differences in the effect of body fat distribution on pulmonary function, some possible explanations may be offered. Sex-based differences in lifestyle factors, hormonal system and pulmonary structure could affect pulmonary function. Another possible mechanism is a difference in how fat distribution associated with weight gain affects the thoracic mechanism in men vs. women, so that the location of fat deposition in women does not adversely affect lung function [24]. Cross-sectional and longitudinal studies of lung function suggest that the effects on respiratory mechanics might be more pronounced in men than in women for any given body fat distribution pattern [9, 11, 27, 28]. It has also been suggested that lung function is influenced by sex differences, perhaps due to a lower functional impairment (smoke-induced) in women smokers, compared to men who smoke [30]; however, large epidemiological studies show that susceptibility to tobacco is similar in both sexes [31].

BMI category in smokers is associated with worse health status and impaired lung function. Recent findings delineate a “U-shaped” association between BMI and extreme weight categories, such that both the obese (BMI ≥30 kg/m^2^) and the lean to underweight (BMI <25 kg/m^2^) smokers had lower FEV1 and worse health status [32]. In our study, the results confirm that overweight and obesity are positively associated with FEV1/FVC ratio in both sexes. Some studies that included measurements of central adiposity have also observed that these tend to correlate with worse lung function, even in non-obese individuals [33]. However, other authors found no significant differences in FEV1/FVC ratio between obese and non-obese individuals [34]. Although the pattern of fat distribution appears to have a more significant influence on FEV1% and FVC% than more commonly used measures of general obesity such as continuous BMI, our results show that BMI >25 kg/m^2^ has a greater direct effect on the FEV1/FVC ratio. This spirometric variable discriminates obstructive ventilation disorder, while a reduction in FVC% accompanies the reduction or maintenance of FEV1%, suggesting a restrictive pattern that can be explained by the alteration in ventilator mechanics experienced by people with obesity [35, 36]. When abdominal fat deposition occurs and BMI increases, the descent of the diaphragm during inspiration is limited, reducing the expiratory reserve volume by displacing the diaphragm upward and reducing functional volume in the thoracic cavity [6, 9, 12]. Another possible mechanism is that chest-wall adiposity may impede expansion and excursion of the rib cage, through a direct loading effect or by altering intercostal muscle function, which decreases inspiratory muscle activity [37, 38]. In addition to these mechanical processes, lung function may also be affected by chronic low-grade inflammatory processes that accompany obesity. It has been shown that excess body fat is associated with markers of systemic and vascular inflammation such as C-reactive protein, interleukin-6, tumour necrosis factor-α, leptin and adiponectin [39]. As a whole, the available data confirm a much more complex relationship between anthropometric changes and lung function than can be ascribed solely to inflammatory effects, and growing evidence suggests that an interaction of adipokine disorder, mechanical disturbances and changes in muscle mass results in a combined effect on lung impairment and its manifestations [40].

## Conclusions

In a Mediterranean population of smokers without respiratory symptoms, abdominal obesity has a negative impact on lung function. Central fat distribution appears to have a stronger relationship with pulmonary functional parameters in men than in women. In addition to BMI, other indexes of fat distribution (WC and WHtR) can be easily obtained during routine clinical practice and can be useful tools to indicate the advisability of carrying out a full assessment. Sensitization of primary care physicians to the identification of smokers with these conditions might increase referrals for lung function testing and lead to earlier diagnosis and appropriate patient management. What is known is that quitting smoking and losing weight are likely the best way to improve lung health in this rapidly growing patient population.
